# Triple Negative Breast Cancers Have a Reduced Expression of DNA Repair Genes

**DOI:** 10.1371/journal.pone.0066243

**Published:** 2013-06-25

**Authors:** Enilze Ribeiro, Monica Ganzinelli, Daniele Andreis, Ramona Bertoni, Roberto Giardini, Stephen B. Fox, Massimo Broggini, Alberto Bottini, Vanessa Zanoni, Letizia Bazzola, Chiara Foroni, Daniele Generali, Giovanna Damia

**Affiliations:** 1 Laboratório de Citogenética Humana e Oncogenética, Departamento de Genética, UFPR, Curitiba, Paraná, Brazil; 2 Laboratory of Molecular Pharmacology, IRCCS – Istituto di Ricerche Farmacologiche Mario Negri, Milan, Italy; 3 U.O.Multidisciplinare di Patologia Mammaria, Laboratorio di Oncologia Molecolare Senologica, A.O. Istituti Ospitalieri di Cremona, Cremona, Italy; 4 U.O.Anatomia Patologica, A.O. Istituti Ospitalieri di Cremona, Cremona, Italy; 5 Peter MacCallum Cancer Centre, East Melbourne, Victoria, Australia; University of Pittsburgh, United States of America

## Abstract

DNA repair is a key determinant in the cellular response to therapy and tumor repair status could play an important role in tailoring patient therapy. Our goal was to evaluate the mRNA of 13 genes involved in different DNA repair pathways (base excision, nucleotide excision, homologous recombination, and Fanconi anemia) in paraffin embedded samples of triple negative breast cancer (TNBC) compared to luminal A breast cancer (LABC). Most of the genes involved in nucleotide excision repair and Fanconi Anemia pathways, and *CHK1* gene were significantly less expressed in TNBC than in LABC. *PARP1* levels were higher in TNBC than in LABC. In univariate analysis high level of *FANCA* correlated with an increased overall survival and event free survival in TNBC; however multivariate analyses using Cox regression did not confirm *FANCA* as independent prognostic factor. These data support the evidence that TNBCs compared to LABCs harbour DNA repair defects.

## Introduction

Triple negative breast cancer (TNBC) defines a clinical subset of breast cancer negative for estrogen receptor (ER), progesterone receptor (PR) and human epidermal growth factor receptor type 2 (HER2). It accounts for about 15% of breast tumors and is characterized by an aggressive clinical course with therapeutic resistance, high rates of local and systemic relapse, and poor survival. This is probably due to the intrinsic biology of this type of tumor as well as to the absence of specific targeted treatments such as hormonal therapy used for ER positive tumors and trastuzumab/lapatinib for HER2 over-expressing tumors [1], [2].

TNBC encompasses more than one subtype [3], [4]. Morphologically they include metaplastic, adenoid cystic and medullary like and at a molecular level they display different mRNA profiles such as basal and claudin type [5], [6]. Cumulating evidence suggest that the triple negative phenotype on clinical assays enriches for basal-like cancer, but no complete overlap exists between the two groups [7], [8]. Indeed, 25% of the TNBCs are not basal-like on gene expression profile and similarly there are basal-like tumors that are not triple negative by immunohistochemistry.

Most of the tumors that develop in women with germline mutations in the *BRCA1* breast cancer susceptibility gene are TNBC [9], [10]. *BRCA1* has a crucial role in the repair of double-strand breaks and its mutation leads to cancer predisposition and genomic instability [11], [12]. *BRCA1* related TNBCs share several pathological features with sporadic TNBCs and cluster within the basal subtype by gene expression profile [13]. Thus it is not surprising that there are data suggesting that a proportion of sporadic basal-like breast cancers may have a dysfunctional *BRCA1* pathway, due to gene promoter methylation or transcriptional inactivation [13], [14]. Deficits in other DNA-repair pathways, such as base excision repair (BER) inactivation [15], MTMG promoter methylation [16] and lack of hOGG1 [17] have been reported in TNBCs. These tumors also exhibit higher DNA copy alterations and loss of heterozygosity than other breast cancer types, suggesting higher genomic instability [18]–[20]. These latter data, together with the reported association between *BRCA1* deficiency and TNBC, suggest that DNA repair alterations may be important for the development of this tumor type. If this is the case, there could have important prognostic and predictive implications, such as the response to the currently used anticancer agents and to novel targeted agents, as the poly-ADP-ribose polymerase (PARP) inhibitors [21].

While tests for the evaluation of DNA repair defects in primary cells or in clinical samples have been described [22], [23], to our knowledge validated functional assays for the quantification of the tumor DNA repair capabilities to be used in clinical setting do not exist. There are however some biomarkers that, even if not completely validated [24]–[27], have been used as surrogate of DNA repair functionality, i.e. the mRNA and/or expression levels of key proteins involved in DNA repair pathways. Aim of our study was to evaluate in a multiparametric manner the mRNA expression level of different key player of DNA repair pathways (i.e. base excision repair-BER-, nucleotide excision repair-NER- and homologous recombination- HR- and Fanconi anemia- FA) in TNBCs compared with LABCs and to correlate with clinico-pathological patient characteristics.

## Materials and Methods

### Patients and samples

150 formalin-fixed paraffin embedded core breast cancer samples, diagnosed as triple negative (80 cases) and luminal A (70 cases), were retrospectively collected from patients who came to the medical observation at the Breast Care Unit, A.O. Istituti Ospitalieri di Cremona, Italy. Ethical permission for the study was obtained from the A.O. Istituti Ospitalieri di Cremona (Italy) ethical committee. Written informed consent was obtained from patients, if applicable. All the samples were anonymized by a pathologist staff member and none of the researcher conducting the gene expression analysis had access to disclosed clinico-pathological data. The patients characteristics are summarized in Table 1.

**Table 1:** Baseline patients' demographic.

CLINICAL PARAMETERS	TNBC (N = 80)	LABC (N = 70)
**Follow-up time –** years		
Median (range)	5.5 (0.1–16.0)	3.16 (0.6–17.3)
Mean ± SD	6.7±4.7	5.9±5.5
**Age –** years		
Median (range)	59.5 (33.1–91.3)	66.6 (36.5–84.5)
Mean ± SD	60.2±14.4	63.1±11.5
**Age** – no. (%)		
<65	46 (57.5)	31 (44.3)
≥65	34 (42.5)	39 (55.7)
**Menopausal status** – no. (%)		
Pre-	19 (23.7)	12 (17.1)
Post-	59 (73.8)	58 (82.9)
not known	2 (2.5)	0 (0.0)
**Clinical tumor size** – no. (%)		
≤20 mm	16 (20.0)	21 (30.0)
>20 mm	36 (45.0)	12 (17.1)
not known	28 (35.0)	37 (52.9)
**Tumor size at surgery** – no. (%)		
≤20 mm	36 (45.0)	42 (60.0)
>20 mm	22 (27.5)	11 (15.7)
not known	22 (27.5)	17 (24.3)
**Pathological T staging** – no. (%)		
T1	41 (51.3)	48 (68.6)
T2	20 (25.0)	11 (15.7)
T3	2 (2.5)	1 (1.4)
T4	4 (5.0)	3 (4.3)
not known	13 (16.2)	7 (10.0)
**Pathological N staging** – no. (%)		
N0	37 (46.3)	66 (94.3)
N+	18 (22.5)	0 (0.0)
not known	25 (31.2)	4 (5.7)
**Metastatic status** – no. (%)		
M0	60 (75.0)	68 (97.1)
M1	4 (5.0)	0 (0.0)
not known	16 (20.0)	2 (2.9)
**Pathological staging** – no. (%)		
Stage I	28 (35.0)	48 (68.6)
Stage IIA	13 (16.3)	11 (15.7)
Stage IIB	5 (6.2)	1 (1.4)
Stage IIIA	4 (5.0)	0 (0.0)
Stage IIIB	4 (5.0)	3 (4.3)
Stage IV	4 (5.0)	0 (0.0)
not known	22 (27.5)	7 (10.0)
**Basal histotype** – no. (%)		
IDC	66 (82.5)	23 (32.9)
ILC	5 (6.2)	16 (22.9)
Other	7 (8.8)	30 (42.8)
not known	2 (2.5)	1 (1.4)
**Basal tumor grade** – no. (%)		
1	1 (1.2)	6 (8.6)
2	14 (17.5)	47 (67.1)
3	64 (80.1)	15 (21.4)
not known	1 (1.2)	2 (2.9)
**Basal Ki67** – no. (%)		
Median (range)	37 (3–90)	7.5 (1–16)
Mean ± SD	44.6±26.4	7.3±3.3
<10	3 (3.7)	50 (71.4)
≥10	75 (93.8)	20 (28.6)
<20	15 (18.7)	70 (100.0)
≥20	63 (78.8)	0 (0.0)
not known	2 (2.5)	0 (0.0)
**Neoadjuvant treatment** – no. (%)		
Done	35 (43.8)	19 (27.2)
not done	38 (47.5)	50 (71.4)
not known	7 (8.7)	1 (1.4)
**Surgical treatment** – no. (%)		
Mastectomy	21 (26.3)	14 (20.0)
Breast conservation	48 (60.0)	54 (77.1)
not known	11 (13.7)	2 (2.9)
**Vital status** – no. (%)		
Alive	53 (66.2)	69 (98.6)
Dead	27 (33.8)	1 (1.4)

SD: standard deviation; IDC: invasive ductal carcinoma; ILC: invasive lobular carcinoma.

### Definition of molecular classes

Tumour grade was evaluated using the modified Bloom and Richardson method. Immunohistochemical evaluation was performed on paraffin-embedded tumour samples obtained at diagnosis. Estrogen receptor, progesterone receptor, HER2 staining were carried out at the Pathology Unit of the A.O. Istituti Ospitalieri di Cremona, Italy. The immunohistochemical method used for routine markers is fully described elsewhere [28]. Tumors were classified as: luminal A (hormone receptor positive: estrogen receptor + and/or progesterone receptor + and HER2–), and triple-negative (ER-ve, PR-ve, HER2-ve) [29].

### RNA and real time

RNA was extracted from core biopses with the High Pure FFPE RNA Micro Kit (Roche). RNA concentrations were then measured by NanoDrop spectrophotometer and retro-transcription to cDNA was performed using High Capacity cDNA Reverse Transcription Kit (Applied Biosystem) in a 96well plate, one for each hystological subgroup. 15ug are preamplified with TaqMan Preamp Master mix (Applied Biosystem) and with the pool of primers used later for real time PCR reactions. Genes selected have a key role in the BER (*PARP1*); in the NER pathway (*ERCC1, XPA, XPD, XPF* and *XPG*); in the FA pathway (*BRCA1, FANCA, FANCC, FANCD2*, *FANCF and PALB2*); in DNA damage checkpoint pathway (*Chk1*). Optimal primer pairs (Table 2) were chosen, spanning splice junctions, using PRIMER-3 software (http://frodo.wi.mit.edu/cgi-bin/primer3/primer3_www.cgi) and the specificity was verified by detecting single-band amplicons of the PCR products. The EP Motion robot (Eppendorf) dispensed 10 ng of cDNA in triplicate on a 384 wells plate, one for each gene and for each subgroup. The reaction was performed with Sybr Green PCR master mix (Applied Biosystem) and the curve of dissociation was evaluated. A standard curve for each specific gene was included in each plate for an absolute quantification of the copy of RNA. Samples were then normalized using the absolute copy number of the housekeeping gene (ciclophilin A). The two different sample plates (TNBC and LABC) were calibrated using inter-run calibrators samples (four different breast cancer cell lines) to correct for run to run technical variation. The calibration procedure was performed on a gene per gene basis.

**Table 2:** Real Time PCR primers.

GENE	PRIMER F	PRIMER R
CICLOPHILIN A	GACCCAACACAAATGGTTCC	TTTCACTTTGCCAAACACCA
PARP1	AAGAAATGCAGCGAGAGCAT	CCAGTGTGGGACTTTTCCAT
ERCC1	GGCCTATGAGCAGAAACCAG	TTCACGGTGGTCAGACATTC
XPA	GAACCACTTTGATTTGCCAAC	TTGTTTTGCCTCTGTTTTGG
XPF	GAGAAATCTTTTTGTGAGGAAACTG	CAACTTCAGGTTTGTGCTGTTC
XPG	AAGCCATCAAAACTGCCTTC	TCGTTTTCTCTTCGAACTTGG
XPD	GTGGCCATCAGCTCCAAAT	CAGCAGGAGGTTCCCATAGT
BRCA1	GCCAGAAAACACCACATCAC	CAGTGTCCGTTCACACACAA
FANCA	GAGGTTCTTCAGTCATACCCTGA	TCTCTCTGCATCTGAACAGCA
FANCC	CCAGCCAGAGTTCTTTGAGG	CGAAGCCAGAGGCAGACTAC
FANCD2	CCCATCTGCTATGATGATGAA	CGTATTTGCTGAGGGGATATG
FANCF	GCTAGTCCACTGGCTTCTGG	GGTGGCGGCTAGTCACTAAA
PALB2	TGGGACCCTTTCTGATCAAC	GGGGCATCAAAAATTGGTTT
CHK1	GGTCACAGGAGAGAAGGAAT	TCTCTGACCATCTGGTTCAGG

### Data and statistical analysis

The Mann-Whitney U test was chosen to compare clinicopathological and gene expression data between the two groups of TNBCs and LABCs. The median is taken as valid proxy for expression level of all genes. Relationships between family gene expression levels were examined using the Spearman's rank correlation. Each gene distribution was split into three expression levels (low, intermediate and high level) defined by the two tertiles calculated on patients who did not die at the end of the study. For further testing, each gene distribution was dichotomized (low and high level) using the median gene expression value as the cut-off point. Consistently, patients were divided into different subgroups based on different gene expression level. Univariate comparisons for all categorical variables were performed by Pearson's chi-squared test with Yates's correction for continuity or Fisher's exact test, when appropriate. Overall survival (OS) and event-free survival (EFS) were classified as outcome measures and defined as the length of time from the start of neoadjuvant chemotherapy or the date of curative surgery (in patients treated with only adjuvant therapy) or the date of diagnosis (in patients diagnosed with metastases) to the last follow-up date or death (irrespective of the cause) or to the first relapse or progression event. OS and EFS curves were plotted with the Kaplan-Meier method. The log-rank test and Cox proportional hazard models were used to compare time-to-event distributions between subgroups. All analyses were performed using SPSS (version 10.0– Chicago, IL) and STATISTICA software system (version 6– Tulsa, OK). All tests were performed two-sided and p values <0.05 were considered statistically significant.

## Results

### Patients' characteristics

We considered a cohort of 150 paraffin embedded core biopsies, 80 with a pathological diagnosis of TNBC and 70 with a diagnosis of LABC that came to the medical attention of a single medical center, the Breast Care Unit of A.O. Istituti Ospitalieri di Cremona from January 5th, 1995 to August 19th, 2008. The two groups (TNBC vs LABC), whose characteristics are depicted in Table 1, were comparable in terms of age (p = 0.2056) and menopausal status at diagnosis (p = 0.4493), surgical treatment (p = 0.3200), pathological T staging at surgery (p = 0.1404), and follow-up time (p = 0.0649).

### mRNA expression of the 13 genes involved in DNA repair pathways

Genes involved in different DNA repair pathways were studied by RT-PCR and Table 3 summarizes the data for both TNBC and LABC. There was considerable variability in expression levels in the individual genes. The levels of the NER genes, *ERCC1*, *XPA, XPF* and *XPD* genes were significantly lower in TNBCs than in LABCs, while there was no difference for *XPG*. FA genes were less expressed than NER genes in TNBC. *BRCA1*, *FANCD2*, *FANCF* and *PALB2* genes were significantly less expressed in TNBCs than in LABCs. *CHK1* gene was significantly less expressed in TNBCs than in LABCs. On the contrary, *PARP1* levels were higher in TNBCs than in LABCs.

**Table 3:** Normalized and calibrated values (mean± SD and median) of the different DNA repair genes in tumor samples.

PATH WAY	GENE	TNBC (N = 80)		LABC (N = 70)		p value*
		Mean ± SD	Median	Mean ± SD	Median	
**BER**	*PARP1*	10.87±10.92	8.250	7.05±5.46	5.221	**0.0002**
**NER**	*ERCC1*	0.71±0.66	0.633	6.87±20.77	1.040	**<0.0001**
	*XPA*	0.10±0.08	0.080	0.16±0.21	0.104	**0.0309**
	*XPF*	23.35±55.61	5.108	68.93±107.05	21.996	**<0.0001**
	*XPG*	0.66±0.71	0.468	1.51±3.52	0.287	0.1534
	*XPD*	0.276±0.360	0.186	6.68±10.26	3.662	**<0.0001**
**FA**	*BRCA1*	0.028±0.025	0.020	0.83±3.36	0.062	**<0.0001**
	*FANCA*	0.138±0.279	0.057	0.27±0.71	0.094	0.8293
	*FANCC*	0.005±0.007	0.003	0.002±0.050	0.003	0.3627
	*FANCD2*	0.08±0.124	0.054	4.45±23.32	0.150	**<0.0001**
	*FANCF*	0.026±0.027	0.018	0.451±1.170	0.031	**<0.0001**
	*PALB2*	0.453±0.563	0.306	5.91±23.03	2.010	**0.0006**
**SEN** **SOR**	*CHK1*	0.179±0.300	0.108	11.51±46.44	1.779	**<0.0001**

* *p* value indicate the difference of the gene expression levels between TNBC anl LABC; *p* value in bold are significant.

### Association between gene expression and clinic-pathological characteristics

Table 4 reports the *p* values found between the gene expression level based on tertiles (low, intermediate and high level as specified in Materials and Methods) and some clinic-pathological characteristics in TNBC. Lower levels of *XPG* and *FANCA* were associated with higher pathological T classification at surgery (pT2-4 vs pT1); higher level of *XPF* was associated with no lymph node involvement (pN0 vs pN1-3), and higher levels of *FANCA* correlated with vital status. Using the median gene expression value as a cut-off point of categorization (low and high level), only the association of *XPG* and *FANCA* expression levels with pT classification remained statistically significant. No other associations were found among the other clinic-pathological evaluated parameters (age at diagnosis-65 years vs ≥65 years-; menopausal status-pre-menopausal vs post-menopausal-; clinical dimension at diagnosis-≤20 mm vs >20 mm-; basal histotype-ductal infiltrating carcinoma vs others-; basal grading-G1-2 vs G3-; basal Ki67-<14% vs ≥14% and <20% vs ≥20%-; clinical complete response-cCR vs not cCR-; pathological complete response-pCR vs not pCR-). When the same analysis was performed in the LABC cohort of patients, none association could be found.

**Table 4:** Significant*p* values for the association between gene expression and clinic-pathological characteristics of triple negative breast cancer patients (univariate analysis, categorization based on tertiles).

PATHWAY	GENE	pT classification	pN classification	Vital status
		at surgery	at surgery	at last follow up
**BER**	*PARP1*			
**NER**	*ERCC1*			
	*XPA*			
	*XPF*		0.0162	
	*XPG*	0.0437		
	*XPD*			
**FA**	*BRCA1*			
	*FANCA*	0.0366		0.0144
	*FANCC*			
	*FANCD2*			
	*FANCF*			
	*PALB2*			
**SENSOR**	*CHK1*			

**Figure 1:**
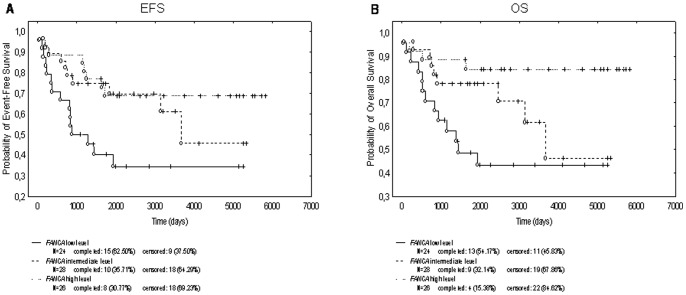
Kaplan-Meier estimates for event-free survival (panel A) and overall survival (panel B) in TNBC patients stratifies by the different level of*FANCA* expression (univariate analysis, categorization based on tertiles).

We correlated the different gene expression levels based on tertiles with OS and EFS. As depicted in Table 5, the intermediate level of *XPG* correlated on univariate analysis with increased OS compared to the low level. Interesting enough, the high level of *FANCA* correlated with an increased OS and EFS compared to the low level. Again, the intermediate level of *FANCA* correlated with an increase in EFS compared to the low level. The Kaplan-Meier curves (Figure 1) shows that higher level of *FANCA* correlated with an increased OS (p = 0.0153) and an increased EFS (p = 0.0207). Furthermore, using the median gene expression value as a cut-off point of categorization, we again found a trend (not significant) toward better OS (p = 0.0906) and EFS (p = 0.0999) in patients with high level of *FANCA*. Multivariate analyses using Cox regression, however, showed that the *FANCA* expression level was not an independent prognostic factor in TNBC patients adjusted for the potential confounding factors: age at diagnosis, surgical treatment, pathological T and N staging at surgery. As regards LABCs, OS and EFS data were not yet mature.

**Table 5:** Overall survival and event-free survival in triple negative breast cancer patients by expression of the different genes (univariate analysis, categorization based on tertiles).

			TNBC (N = 80)	
PATHWAY	GENE	EXPRESSION	OS	EFS
			*p* value	*p* value
**BER**	***PARP1***	II-I terziles	0.3056	0.2334
		III-I terziles	0.6140	0.4632
**NER**	***ERCC1***	II-I terziles	0.9993	0.9450
		III-I terziles	0.1868	0.3955
	***XPA***	II-I terziles	0.2035	0.0957
		III-I terziles	0.7071	0.9908
	***XPF***	II-I terziles	0.7813	0.9566
		III-I terziles	0.1088	0.4704
	***XPG***	II-I terziles	**0.0344**	0.0674
		III-I terziles	0.2870	0.2902
	***XPD***	II-I terziles	0.1010	0.0764
		III-I terziles	0.1403	0.2480
**FA**	***BRCA1***	II-I terziles	0.4812	0.6162
		III-I terziles	0.4196	0.5115
	***FANCA***	II-I terziles	0.1088	**0.0408**
		III-I terziles	**0.0045**	**0.0141**
	***FANCC***	II-I terziles	0.3579	0.5383
		III-I terziles	0.0850	0.0736
	***FANCD2***	II-I terziles	0.0845	0.1352
		III-I terziles	0.1554	0.2216
	***FANCF***	II-I terziles	0.7556	0.9254
		III-I terziles	0.6192	0.7587
	***PALB2***	II-I terziles	0.5170	0.5381
		III-I terziles	0.2010	0.1617
	***PARP1***	II-I terziles	0.3056	0.2334
		III-I terziles	0.6140	0.4632
**SENSOR**	***CHK1***	II-I terziles	0.7740	0.4396
		III-I terziles	0.2325	0.2601

The mRNA expression distribution of all the genes was split into three groups, as described in Materials and Methods. *p* value in bold are significant.

## Discussion

TNBC represents a subset of breast cancer with well documented poor prognosis, whose real causes are still to be defined even if a number of adverse factors have been advocated, such as the fact that these tumors are at the diagnosis commonly of high nuclear mitotic grade, of larger tumor size and with a more aggressive expression profile (low BCL2 and high p53 and Ki67 expression) [5], [30], [31]. Several studies have demonstrated significant lower EFS in patients with TNBC compared to patients with non-TNBC; in addition, shorter OS in TNBC patients is widely reported [3], [32]–[34]. Despite the poor prognosis, these tumors are particularly chemosensitive at least in a short-term time frame [35], [36]. In fact, patients with TNBC have increased pathologic complete response (pCR) rates compared to non-TNBC patients after treatment with taxanes and antracycline agents. Wang at al reported a 38% of pCR in TNBC and 14% in non-TNBC patients treated with taxane in combination with antracycline [37]. Better response rates were also reported after treatment with alkylating agents, including cyclophosphamide, cisplatin, and carboplatin [38].

TNBC have been shown to harbour DNA repair deficiencies, including *BRCA1* dysfunction, due to promoter methylation or deregulation of other genes involved in their transcriptional regulation [9], BER inactivation [15], MTMG promoter hypermethylation [16] and lack of hOGG1 [17]. Given the importance of the cellular DNA repair in determining the cellular response to different anticancer agents [39]–[43], *a priori* knowledge of the repair status of a given tumor could play an important role in selection of the most appropriate therapy. Attempts to investigate the functionality of a given DNA repair pathway have been undertaken and shown to be feasible. Willers et al reported the successful detection of *BRCA1*, *FANCD2* and *RAD51* foci in seven breast cancer biopsies irradiated *ex vivo*, suggesting the possibility to detect defects within the complex FA/BRCA DNA damage response pathway [44]. Isolation of epithelial cells from breast tumor specimens and application of specific functional DNA repair assay systems led to the detection of specific defects in double strand breaks repair [22], while homologous recombination status could be determined in ovarian cancer samples by *RAD51* foci formation after *in vitro* treatment with a PARP inhibitor [23]. However, all these assays still need to be technically improved (ie. requirement for an automated foci scoring and setting up of tumor specific primary cultures) rendering difficult their wide spread clinical use. An alternative approach is the study of biomarkers that, even if not completely validated [24]–[27], can be considered surrogate of DNA repair functionality, i.e. the mRNA and/or expression levels of key proteins involved in DNA repair pathways.

The expression profile of a number of genes involved in the different DNA repair pathways was studied in a cohort of TNBC samples as a surrogate markers of DNA repair status and correlated with different clinic-pathological characteristics, including survival. These data were compared to the ones obtained in a group of patients with LABC, that represent the breast cancer subtype with the best prognosis.

The levels of expression of all the genes involved in the NER and FA pathways were significantly lower in TNBC as compared to LABC samples, with the exception of *XPG*, *FANCA* and *FANCC* whose difference did not reach a statistically significant value.

These expression data might in part explain the extremely high sensitivity of TNBC patients to chemotherapy. It has in fact been demonstrated that not only the deficiency, but also low levels of mRNA/protein involved in NER, i.e *ERCC1* and *XPF* or *FA* genes are associated with increased sensitivity to alkylating agents in different cellular systems [45]. At the same time, the low expression level of these genes might explain the bad prognosis of the tumors, even if they do respond well to chemotherapy. The low DNA repair capacity would in fact increase the genomic instability of the tumor cells enabling them to accumulate much more mutations through which cells would became more aggressive. Sporadic stage I breast cancer have recently been reported to exhibite a significant deficiency of NER capacity relative to epithelial control tissue [46] by both functional unscheduled DNA synthesis and mRNA expression of NER genes. Low expression of 4 proteins – XPF, FANCD2, MLH1 and pMK2– assessed in FFPE tumor specimens by semiquantitative immunohistochemistry, was associated with shorter recurrence free survival in multivariate analysis [47], corroborating the idea that tumors with reduced DNA repair capacity will have a higher degree of genomic instability and therefore behave more aggressively.

The data on *FANCA* are, on the contrary, counter-intuitive and intriguing. *FANCA* was not differentially expressed between TNBC and LABC, but it was found to positively correlate in univariate analysis with both EFS and OS (higher level correlated with longer EFS and OS). *FANCA* is one of the 13 proteins cooperating in a common DNA repair pathway (for rev see [48], [49]). *FANCA,* higher expression correlated with an improved outcome that could be a consequence of a less malignant phenotype, due to a more stable tumor genome. This resembles what observed for the expression of *ERCC1* in non small cell lung cancer (NSCLC), where it has been shown that higher *ERCC1* levels were correlated with a better prognosis [50], [51], but was also higher levels of *ERCC1* were a marker of resistance to a platinum containing regimens [50]. Recently, the experimental finding that *ERCC1* negative NSLC samples display a higher genomic instability corroborate the more unstable and then the worst clinical history of *ERCC1* low/negative tumors [52].

There are few, but contrasting data, on *CHK1* expression in different breast cancer subtypes. CHK1 is a kinase that has an important role in both the maintenance of genomic instability and in the transduction pathways after DNA damage [53]. Verlinden et al. [54] reported a twofold higher expression (as assessed by RT-PCR) of *CHK1* in grade 3 TNBC than in other grade 3 tumor breast subtype; while no difference CHK1 protein expression was found in different breast cancer cell lines. These discrepancies can be related to the different methodologies used (RT-PCR vs western blotting; relative vs absolute RT-PCR values), samples size and the pathological classification of the different tumor analyzed. All tumor samples analyzed expressed *PARP1* mRNA, whose levels were higher in TNBC compared to LABC, reinforcing the data that neoplastic tumors express high level of PARP [55], [56].

There are a number of issues that limits the present study: i.e. its retrospective nature, the relative small sample size and the fact it analysed the mRNA expression levels of DNA repair protein rather than the quantification of a functional DNA repair status. Nevertheless, our data are the first to show the mRNA expression of multiple DNA repair genes involved in three different DNA repair pathways (BER, NER and FA) in a cohort of TNBC and LABC.

These data support the experimental evidence that TNBC harbour DNA repair defects and this could explain both the extremely chemo-sensitivity of this tumor type to chemotherapy in a short term and the worse outcome probably correlated with the inability to counteract the increased genomic instability for the lack of an efficient DNA damage status.

## References

[1] Carey LA (2011) Directed therapy of subtypes of triple-negative breast cancer. Oncologist 16 Suppl 171–78. 10.1634/theoncologist.2011-S1-71 21278443

[2] Hudis CA, Gianni L (2011) Triple-negative breast cancer: an unmet medical need. Oncologist 16 Suppl 11–11. 10.1634/theoncologist.2011-S1-01 21278435

[3] de Ruijter TC, Veeck J, de Hoon JP, van Engeland M, Tjan-Heijnen VC (2011) Characteristics of triple-negative breast cancer. J Cancer Res Clin Oncol 137: 183–192. 21069385 10.1007/s00432-010-0957-x PMC3018596

[4] Chacon RD, Costanzo MV (2010) Triple-negative breast cancer. Breast Cancer Res 12 Suppl 2S3. 10.1186/bcr2574 PMC2972557 21050424

[5] Perou CM (2011) Molecular stratification of triple-negative breast cancers. Oncologist 16 Suppl 161–70. 10.1634/theoncologist.2011-S1-61 21278442

[6] Perou CM, Sorlie T, Eisen MB, van de Rijn M, Jeffrey SS, et al (2000) Molecular portraits of human breast tumours. Nature 406: 747–752. 10963602 10.1038/35021093

[7] Bertucci F, Finetti P, Cervera N, Esterni B, Hermitte F, et al (2008) How basal are triple-negative breast cancers? Int J Cancer 123: 236–240. 18398844 10.1002/ijc.23518

[8] Rakha EA, Elsheikh SE, Aleskandarany MA, Habashi HO, Green AR, et al (2009) Triple-negative breast cancer: distinguishing between basal and nonbasal subtypes. Clin Cancer Res 15: 2302–2310. 19318481 10.1158/1078-0432.CCR-08-2132

[9] Turner NC, Reis-Filho JS (2006) Basal-like breast cancer and the BRCA1 phenotype. Oncogene 25: 5846–5853. 16998499 10.1038/sj.onc.1209876

[10] Atchley DP, Albarracin CT, Lopez A, Valero V, Amos CI, et al (2008) Clinical and pathologic characteristics of patients with BRCA-positive and BRCA-negative breast cancer. J Clin Oncol 26: 4282–4288. 18779615 10.1200/JCO.2008.16.6231 PMC6366335

[11] Zhang J, Powell SN (2005) The role of the BRCA1 tumor suppressor in DNA double-strand break repair. Mol Cancer Res 3: 531–539. 16254187 10.1158/1541-7786.MCR-05-0192

[12] Hartman AR, Ford JM (2002) BRCA1 induces DNA damage recognition factors and enhances nucleotide excision repair. Nat Genet 32: 180–184. 12195423 10.1038/ng953

[13] Turner N, Tutt A, Ashworth A (2004) Hallmarks of ‘BRCAness’ in sporadic cancers. Nat Rev Cancer 4: 814–819. 15510162 10.1038/nrc1457

[14] Turner NC, Reis-Filho JS, Russell AM, Springall RJ, Ryder K, et al (2007) BRCA1 dysfunction in sporadic basal-like breast cancer. Oncogene 26: 2126–2132. 17016441 10.1038/sj.onc.1210014

[15] Alli E, Sharma VB, Sunderesakumar P, Ford JM (2009) Defective repair of oxidative dna damage in triple-negative breast cancer confers sensitivity to inhibition of poly(ADP-ribose) polymerase. Cancer Res 69: 3589–3596. 19351835 10.1158/0008-5472.CAN-08-4016 PMC2681413

[16] Fumagalli C, Pruneri G, Possanzini P, Manzotti M, Barile M, et al.. (2012) Methylation of O (6)-methylguanine-DNA methyltransferase (MGMT) promoter gene in triple-negative breast cancer patients. Breast Cancer Res Treat. 10.1007/s10549-011-1945-9 22228432

[17] Karihtala P, Kauppila S, Puistola U, Jukkola-Vuorinen A (2012) Absence of the DNA repair enzyme human 8-oxoguanine glycosylase is associated with an aggressive breast cancer phenotype. Br J Cancer 106: 344–347. 22108520 10.1038/bjc.2011.518 PMC3261678

[18] Weigman VJ, Chao HH, Shabalin AA, He X, Parker JS, et al.. (2011) Basal-like Breast cancer DNA copy number losses identify genes involved in genomic instability, response to therapy, and patient survival. Breast Cancer Res Treat [Epub ahead of print]. 10.1007/s10549-011-1846-y PMC3387500 22048815

[19] Bergamaschi A, Kim YH, Wang P, Sorlie T, Hernandez-Boussard T, et al (2006) Distinct patterns of DNA copy number alteration are associated with different clinicopathological features and gene-expression subtypes of breast cancer. Genes Chromosomes Cancer 45: 1033–1040. 16897746 10.1002/gcc.20366

[20] Andre F, Job B, Dessen P, Tordai A, Michiels S, et al (2009) Molecular characterization of breast cancer with high-resolution oligonucleotide comparative genomic hybridization array. Clin Cancer Res 15: 441–451. 19147748 10.1158/1078-0432.CCR-08-1791

[21] Rowe BP, Glazer PM (2010) Emergence of rationally designed therapeutic strategies for breast cancer targeting DNA repair mechanisms. Breast Cancer Res 12: 203. 20459590 10.1186/bcr2566 PMC2879573

[22] Keimling M, Kaur J, Bagadi SA, Kreienberg R, Wiesmuller L, et al (2008) A sensitive test for the detection of specific DSB repair defects in primary cells from breast cancer specimens. Int J Cancer 123: 730–736. 18491400 10.1002/ijc.23551

[23] Mukhopadhyay A, Elattar A, Cerbinskaite A, Wilkinson SJ, Drew Y, et al (2010) Development of a functional assay for homologous recombination status in primary cultures of epithelial ovarian tumor and correlation with sensitivity to poly(ADP-ribose) polymerase inhibitors. Clin Cancer Res 16: 2344–2351. 20371688 10.1158/1078-0432.CCR-09-2758

[24] Lips EH, Mulder L, Hannemann J, Laddach N, Vrancken Peeters MT, et al (2011) Indicators of homologous recombination deficiency in breast cancer and association with response to neoadjuvant chemotherapy. Ann Oncol 22: 870–876. 20937646 10.1093/annonc/mdq468

[25] Rothschild SI, Gautschi O, Lara PN Jr, Mack PC, Gandara DR (2011) Biomarkers of DNA repair and related pathways: significance in non-small cell lung cancer. Curr Opin Oncol 23: 150–157. 21119513 10.1097/CCO.0b013e328341ee38

[26] Konstantinopoulos PA, Spentzos D, Karlan BY, Taniguchi T, Fountzilas E, et al (2010) Gene expression profile of BRCAness that correlates with responsiveness to chemotherapy and with outcome in patients with epithelial ovarian cancer. J Clin Oncol 28: 3555–3561. 20547991 10.1200/JCO.2009.27.5719 PMC2917311

[27] Dienstmann R, Vilar E, Tabernero J (2011) Molecular predictors of response to chemotherapy in colorectal cancer. Cancer J 17: 114–126. 21427555 10.1097/PPO.0b013e318212f844

[28] Generali D, Buffa FM, Berruti A, Brizzi MP, Campo L, et al (2009) Phosphorylated ERalpha, HIF-1alpha, and MAPK signaling as predictors of primary endocrine treatment response and resistance in patients with breast cancer. J Clin Oncol 27: 227–234. 19064988 10.1200/JCO.2007.13.7083

[29] Kornegoor R, Verschuur-Maes AH, Buerger H, Hogenes MC, de Bruin PC, et al.. (2011) Molecular subtyping of male breast cancer by immunohistochemistry. Mod Pathol. 10.1038/modpathol.2011.174 22056953

[30] Irvin WJ Jr, Carey LA (2008) What is triple-negative breast cancer? Eur J Cancer 44: 2799–2805. 19008097 10.1016/j.ejca.2008.09.034

[31] Nishimura R, Arima N (2008) Is triple negative a prognostic factor in breast cancer? Breast Cancer 15: 303–308. 18369692 10.1007/s12282-008-0042-3

[32] Dent R, Trudeau M, Pritchard KI, Hanna WM, Kahn HK, et al (2007) Triple-negative breast cancer: clinical features and patterns of recurrence. Clin Cancer Res 13: 4429–4434. 17671126 10.1158/1078-0432.CCR-06-3045

[33] Kaplan HG, Malmgren JA (2008) Impact of triple negative phenotype on breast cancer prognosis. Breast J 14: 456–463. 18657139 10.1111/j.1524-4741.2008.00622.x

[34] Rhee J, Han SW, Oh DY, Kim JH, Im SA, et al (2008) The clinicopathologic characteristics and prognostic significance of triple-negativity in node-negative breast cancer. BMC Cancer 8: 307. 18947390 10.1186/1471-2407-8-307 PMC2577689

[35] Carey LA, Dees EC, Sawyer L, Gatti L, Moore DT, et al (2007) The triple negative paradox: primary tumor chemosensitivity of breast cancer subtypes. Clin Cancer Res 13: 2329–2334. 17438091 10.1158/1078-0432.CCR-06-1109

[36] Hugh J, Hanson J, Cheang MC, Nielsen TO, Perou CM, et al (2009) Breast cancer subtypes and response to docetaxel in node-positive breast cancer: use of an immunohistochemical definition in the BCIRG 001 trial. J Clin Oncol 27: 1168–1176. 19204205 10.1200/JCO.2008.18.1024 PMC2667821

[37] Wang S, Yang H, Tong F, Zhang J, Yang D, et al (2009) Response to neoadjuvant therapy and disease free survival in patients with triple-negative breast cancer. Gan To Kagaku Ryoho 36: 255–258. 19223741

[38] Tan AR, Swain SM (2008) Therapeutic strategies for triple-negative breast cancer. Cancer J 14: 343–351. 19060597 10.1097/PPO.0b013e31818d839b

[39] Damia G, D'Incalci M (2010) Genetic instability influences drug response in cancer cells. Curr Drug Targets 11: 1317–1324. 20840074 10.2174/1389450111007011317

[40] Damia G, D'Incalci M (2007) Targeting DNA repair as a promising approach in cancer therapy. Eur J Cancer 43: 1791–1801. 17588740 10.1016/j.ejca.2007.05.003

[41] Damia G, Imperatori L, Stefanini M, D'Incalci M (1996) Sensitivity of CHO mutant cell lines with specific defects in nucleotide excision repair to different anti-cancer agents. Int J Cancer 66: 779–783. 8647649 10.1002/(SICI)1097-0215(19960611)66:6<779::AID-IJC12>3.0.CO;2-Z

[42] Tutt AN, Lord CJ, McCabe N, Farmer H, Turner N, et al (2005) Exploiting the DNA repair defect in BRCA mutant cells in the design of new therapeutic strategies for cancer. Cold Spring Harb Symp Quant Biol 70: 139–148. 16869747 10.1101/sqb.2005.70.012

[43] Kennedy RD, D'Andrea AD (2006) DNA repair pathways in clinical practice: lessons from pediatric cancer susceptibility syndromes. J Clin Oncol 24: 3799–3808. 16896009 10.1200/JCO.2005.05.4171

[44] Willers H, Taghian AG, Luo CM, Treszezamsky A, Sgroi DC, et al (2009) Utility of DNA repair protein foci for the detection of putative BRCA1 pathway defects in breast cancer biopsies. Mol Cancer Res 7: 1304–1309. 19671671 10.1158/1541-7786.MCR-09-0149 PMC4239295

[45] Gossage L, Madhusudan S (2007) Current status of excision repair cross complementing-group 1 (ERCC1) in cancer. Cancer Treat Rev 33: 565–577. 17707593 10.1016/j.ctrv.2007.07.001

[46] Latimer JJ, Johnson JM, Kelly CM, Miles TD, Beaudry-Rodgers KA, et al (2010) Nucleotide excision repair deficiency is intrinsic in sporadic stage I breast cancer. Proc Natl Acad Sci U S A 107: 21725–21730. 21118987 10.1073/pnas.0914772107 PMC3003008

[47] Alexander BM, Sprott K, Farrow DA, Wang X, D'Andrea AD, et al (2010) DNA repair protein biomarkers associated with time to recurrence in triple-negative breast cancer. Clin Cancer Res 16: 5796–5804. 21138871 10.1158/1078-0432.CCR-10-0292 PMC4220607

[48] Deans AJ, West SC (2011) DNA interstrand crosslink repair and cancer. Nat Rev Cancer 11: 467–480. 21701511 10.1038/nrc3088 PMC3560328

[49] Kee Y, D'Andrea AD (2010) Expanded roles of the Fanconi anemia pathway in preserving genomic stability. Genes Dev 24: 1680–1694. 20713514 10.1101/gad.1955310 PMC2922498

[50] Olaussen KA, Dunant A, Fouret P, Brambilla E, Andre F, et al (2006) DNA repair by ERCC1 in non-small-cell lung cancer and cisplatin-based adjuvant chemotherapy. N Engl J Med 355: 983–991. 16957145 10.1056/NEJMoa060570

[51] Simon GR, Sharma S, Cantor A, Smith P, Bepler G (2005) ERCC1 expression is a predictor of survival in resected patients with non-small cell lung cancer. Chest 127: 978–983. 15764785 10.1378/chest.127.3.978

[52] Friboulet L, Barrios-Gonzales D, Commo F, Olaussen KA, Vagner S, et al (2011) Molecular Characteristics of ERCC1-Negative versus ERCC1-Positive Tumors in Resected NSCLC. Clin Cancer Res 17: 5562–5572. 21750204 10.1158/1078-0432.CCR-11-0790

[53] Carrassa L, Damia G (2011) Unleashing Chk1 in cancer therapy. Cell Cycle 10: 2121–2128. 21610326 10.4161/cc.10.13.16398

[54] Verlinden L, Vanden Bempt I, Eelen G, Drijkoningen M, Verlinden I, et al (2007) The E2F-regulated gene Chk1 is highly expressed in triple-negative estrogen receptor/progesterone receptor/HER-2 breast carcinomas. Cancer Res 67: 6574–6581. 17638866 10.1158/0008-5472.CAN-06-3545

[55] Domagala P, Huzarski T, Lubinski J, Gugala K, Domagala W (2011) PARP-1 expression in breast cancer including BRCA1-associated, triple negative and basal-like tumors: possible implications for PARP-1 inhibitor therapy. Breast Cancer Res Treat 127: 861–869. 21409392 10.1007/s10549-011-1441-2

[56] Ossovskaya V, Koo IC, Kaldjian EP, Alvares C, Sherman BM (2010) Upregulation of Poly (ADP-Ribose) Polymerase-1 (PARP1) in Triple-Negative Breast Cancer and Other Primary Human Tumor Types. Genes Cancer 1: 812–821. 21779467 10.1177/1947601910383418 PMC3092251

